# Healthcare Providers’ Perceptions and Multi-Level Determinants of Adoption of an AI-Powered Electrocardiography Interpretation Clinical Decision Support System in Ethiopia: A Formative Qualitative Study

**DOI:** 10.3390/ijerph23040513

**Published:** 2026-04-16

**Authors:** Minyahil Tadesse Boltena, Ziad El-Khatib, Amare Zewdie, Paul Springer, Abraham Tekola Gebremedhn, Tsegab Alemayehu Bukate, Yeabsira Alemu Fantaye, Gelan Ayana, Abraham Sahilemichael Kebede, Jude Kong

**Affiliations:** 1 Artificial Intelligence Innovation Lab, Armauer Hansen Research Institute, Ministry of Health, Addis Ababa P.O. Box 1005, Ethiopia; amarezewdie23@gmail.com (A.Z.); abratekola@gmail.com (A.T.G.); tsegab.alemayehu@aau.edu.et (T.A.B.); yeabsiraalemu34@gmail.com (Y.A.F.); 2 Cochrane Ethiopia, Center of Excellence for Evidence Synthesis and Knowledge Translation, Armauer Hansen Research Institute, Ministry of Health, Addis Ababa P.O. Box 1005, Ethiopia; 3 Ethiopian Evidence-Based Health Care Centre, Health Behavior and Society Department, Faculty of Public Health, Institute of Health, Jimma University, Jimma P.O. Box 378, Ethiopia; 4 Department of Global Public Health, Karolinska Institute, 17177 Stockholm, Sweden; ziad.el-khatib@ki.se; 5 MI4People gGmbH, Maxhofstraße 76, 81475 Munich, Germany; paul.springer@mi4people.org; 6 School of Biomedical Engineering, Institute of Technology, Jimma University, Jimma P.O. Box 378, Ethiopia; gelan.ayana@ju.edu.et; 7 Artificial Intelligence and Mathematical Modelling Lab, Dalla Lana School of Public Health, University of Toronto, Toronto, ON M5S 2E4, Canada; jude.kong@utoronto.ca; 8 Lero SFI Research Centre for Software, Health Research Institute, University of Limerick, V94 NYD3 Limerick, Ireland; abraham.kebede@ul.ie; 9 Department of Mathematics, University of Toronto, Bahen Centre for Information Technology, Room 6291, 40 St. George Street, Toronto, ON M5S 2E4, Canada; 10 Africa-Canada Artificial Intelligence and Data Innovation Consortium (ACADIC), York University, 4700 Keele Street, Toronto, ON M3J 1P3, Canada; 11 ⁠Global South Artificial Intelligence for Pandemic and Epidemic Preparedness and Response Network (AI4PEP), 4700 Keele Street, Toronto, ON M3J 1P3, Canada

**Keywords:** artificial intelligence, electrocardiography, cardiovascular disease, healthcare providers, Ethiopia

## Abstract

**Highlights:**

**Public health relevance—How does this work relate to a public health issue?**

- **Addressing the diagnostic gap in the epidemiological transition:** This study characterizes systemic barriers to early cardiovascular disease (CVD) detection, providing empirical evidence that an AI-powered ECG interpretation support system (CDSS) can decentralize specialized diagnostics. The findings illustrate how this technology addresses the scarcity of paper-based interpretation expertise, facilitating equitable access to cardiac screening in resource-limited settings.
- **Augmenting primary healthcare through task-shifting:** The qualitative analysis explored how AI-powered ECG interpretation CDSS serves as a transformative catalyst for task-shifting specialized diagnostic services to the primary level. Participants identified that by reducing subjectivity and simplifying clinical workflows, it enables frontline workers to initiate timely interventions, directly supporting the goal of Universal Health Coverage (UHC).

**Public health significance—Why is this work of significance to public health?**

- **Informing provider-led digital transformation:** This research identified the critical determinants of provider trust, such as the demand for 99% accuracy and rigorous evidence-based validation for AI-powered ECG interpretation CDSS. By synthesizing perspectives from diverse healthcare workers (cardiac nurses, critical care nurses, general practitioners, critical care specialists, and cardiologists), the study establishes a behavioral blueprint for ensuring AI tools are professionally accepted within the Human Resources for Health (HRH) framework.
- **Expert consensus on resource optimization:** The findings reveal that high-level clinicians perceive AI-enabled ECG interpretation as a vital tool for reducing unnecessary referrals and optimizing the allocation of scarce resources. By detecting subclinical pathologies and standardizing readings, the tool is seen as a means to strengthen health system resilience across all tiers of care.

**Public health implications—What are the key implications or messages for practitioners, policymakers, and/or researchers in public health?**

- **Implications for evidence-informed policy:** Successful national scale-up requires a multidimensional readiness framework that coordinates phased deployment with strategic material resource procurement (stable electricity, high-speed internet, digital ECG machines, and access to AI-powered ECG interpretation CDSS). The study provides policy guidance insights for context-specific governance, emphasizing that leadership engagement and sustainable financing are prerequisites for integrating AI into the national health architecture.
- **Implications for evidence-based clinical practice:** To ensure long-term clinical utility and professional autonomy, adoption should be supported by practical, simulation-based training that frames AI as a supportive adjunct rather than a clinician replacement. Clinicians highlighted that models must be calibrated to local epidemiological contexts and integrated with electronic medical records (EMRs) to foster a truly resilient and responsive cardiovascular care network.

**Abstract:**

Cardiovascular diseases (CVDs) are a leading cause of morbidity and mortality globally, with low-resource settings, including Ethiopia facing challenges due to limited early diagnostic services. AI-powered electrocardiography (ECG) interpretation has the potential to improve diagnostic accuracy, decentralize care, and support timely clinical decisions, but evidence on healthcare providers’ perspectives and adoption determinants is limited. This exploratory descriptive qualitative study employed 31 in-depth interviews with healthcare providers. Healthcare providers (cardiologists, internists, cardiac and critical care nurses, critical care specialists, and general practitioners) were purposively selected through maximum variation sampling from ten hospitals in four regions of Ethiopia. Data were transcribed verbatim, coded inductively, and analyzed thematically. The data analysis identified six themes: perceived benefit of AI-powered ECG interpretation CDSS, trust development, workflow integration, ethical concerns, functionality, and adoption determinants. Participants emphasized AI’s potential to enhance accessibility, consistency, and diagnostic accuracy while reducing subjectivity and unnecessary referrals. Acceptance relied on high accuracy, reliable data, and rigorous validation, with the technology seen as supportive rather than replacing clinicians. Material resources, human resource readiness, and leadership engagement were key factors for adoption. Recommendations included phased implementation, continuous training, and model expansion to ensure sustainability and clinical utility. The AI-powered ECG interpretation CDSS was viewed as a valuable adjunct for strengthening cardiovascular care in Ethiopia, highlighting the need for context-sensitive strategies, ethical safeguards, and multi-level system readiness for successful adoption.

## 1. Introduction

Cardiovascular diseases (CVDs) are a leading cause of morbidity and mortality globally, with an increasing burden in low- and middle-income countries (LMICs) such as Ethiopia [1,2]. Timely diagnosis and intervention are critical to reducing adverse outcomes, yet resource constraints, limited specialist availability, and delayed diagnostic processes challenge effective CVD management [3]. Electrocardiography (ECG) remains a fundamental diagnostic tool, but its interpretation requires highly trained healthcare providers, who are often scarce outside tertiary healthcare centers [4].

Advances in artificial intelligence (AI) offer potential solutions to these gaps, particularly through AI-powered ECG reading tools that automate interpretation and support clinical decision-making. A review of the evidence indicates that combining continuous cardiac monitoring with AI-driven predictive analytics, implemented across various global settings, has been effective in detecting early clinical deterioration while reducing diagnostic variability [5]. Such technologies can improve diagnostic accuracy, reduce inter-observer variability, and enable decentralized care, thereby addressing disparities in specialist access [6,7]. Healthcare providers’ perceptions and readiness are critical determinants of successful AI implementation [8]. Trust in the technology, alignment with existing workflows, and ethical acceptability influence adoption at the frontline [9]. Previous studies suggest that clinicians are more likely to use AI tools when they complement, rather than replace, human expertise, highlighting the importance of understanding the healthcare provider’s views on the technology before large-scale deployment [10].

Implementation of AI in resource-limited settings also faces systemic challenges, including infrastructure constraints, human resource capacity, and governance or policy gaps [11]. Reliable electricity, stable internet connectivity, and availability of compatible devices are prerequisites for operational feasibility. Furthermore, sustainable adoption depends on training, leadership support, continuous monitoring, and integration into health information systems, which are often underdeveloped in lower-resource healthcare contexts [12].

Emerging evidence highlights the potential of AI-powered ECG interpretation CDSS to support task-shifting in health settings with limited specialists, allowing non-cardiologists and nurses to deliver timely and accurate cardiovascular assessments. By facilitating rapid interpretation and early detection of critical conditions, AI can enhance patient-centered care and reduce unnecessary referrals. However, adoption depends not only on technological performance but also on user confidence, perceived usefulness, and alignment with local clinical workflows [13]. Despite global progress, evidence on AI adoption and its real-world feasibility in low-resource healthcare settings remains limited [14], underscoring the need for formative studies to explore these multi-level determinants.

Given these considerations, exploring healthcare providers’ perceptions, expectations, and practical experiences is essential to inform context-sensitive strategies for AI-powered ECG interpretation CDSS adoption in Ethiopia, where ECG interpretation capacity remains limited, particularly in rural healthcare settings, and access to trained clinicians and cardiology expertise is scarce. Understanding facilitators and barriers at individual, organizational, and system levels can guide effective implementation, enhance patient care, and maximize the clinical utility of AI interventions in cardiovascular health.

## 2. Materials and Methods

### 2.1. Study Setting and Context

This formative qualitative study was conducted between September and October 2025 across ten selected referral and specialized hospitals in Ethiopia, including facilities in Addis Ababa, Amhara, Oromia, and the central Ethiopia regions. An AI-powered ECG CDSS has been in the pilot implementation phase in ten hospitals in Ethiopia for the past year, and preparations for a nationwide scale-up are underway in the upcoming year. Five of the hospitals included in this study were currently using the technology as a pilot, and four were planned to be included in the upcoming scale-up phase. This approach was intended to capture practical experiences, real-world challenges, and lessons from the pilot and broader perceptions, expectations, and healthcare providers’ insights from the newly included. Hospitals were purposefully selected to include referral and specialized facilities, which were the main centers for CVD care and were located in different parts of the country. This selection aimed to capture diverse real-world contexts for cardiovascular disease (CVD) care and to explore healthcare providers’ perceptions and experiences regarding AI-powered ECG interpretation CDSS adoption at point-of-care.

### 2.2. Study Design

An exploratory descriptive qualitative design was employed. This design enabled the capture of healthcare providers’ perceptions regarding AI-powered ECG reading and perspectives on multi-level adoption determinants of AI-powered ECG interpretation CDSS, including perceived utility, clinical workflow integration, trust, ethical considerations, and contextual feasibility. By focusing on both individual and system-level factors, the study sought to generate actionable insights for sustainable AI implementation in resource-limited settings.

### 2.3. Study Participants

Study participants were healthcare providers directly involved in cardiovascular care, including cardiologists, internists, cardiac nurses, anesthesiologists, and general practitioners. Maximum variation purposive sampling was used to ensure diversity in specialty, years of experience, and level of clinical responsibility. Participants had an average of 6.4 years of experience in CVD care, allowing rich perspectives on frontline adoption challenges and facilitators. Inclusion criteria emphasized direct involvement in ECG interpretation or decision-making related to cardiovascular diagnostics. Potential participants were identified with the assistance of hospital focal persons and department heads and were approached in person by the research team. They were provided with information about the study’s purpose and invited to participate voluntarily. A total of 31 participants were interviewed, with data collection continuing until thematic saturation was achieved.

### 2.4. Data Collection Tool and Procedure

Data were collected through semi-structured in-depth interviews (IDIs) using an interview guide developed specifically for this study. Interviews explored themes, including perceived benefits of AI-powered ECG interpretation CDSS, trust-building, clinical workflow integration, ethical considerations, functionality expectations, adoption determinants, and recommendations for sustainable implementation. Interviews were conducted in participants’ usual work settings, such as consultation rooms and offices, to ensure contextual relevance, and averaged 30–60 min in duration. All interviews were audio-recorded with consent and supplemented with detailed field notes capturing nonverbal cues and environmental context. Interviews were conducted by experienced and trained qualitative data collectors, all of whom had prior experience in qualitative research but no pre-existing professional relationships with the participants.

### 2.5. Rigor and Trustworthiness

To ensure methodological rigor, the study adhered to the principles of credibility, dependability, confirmability, and transferability. The interview guide was pretested in non-study hospitals to refine clarity and relevance. Experienced qualitative researchers conducted, transcribed, and analyzed the interviews to contextualize verbal and nonverbal responses. Member checking and team discussions validated emerging themes, enhancing credibility. An audit trail, including recordings, transcripts, and analytic memos, reinforced dependability and confirmability. The diversity of participants across hospital types and specialties strengthened the transferability of findings to similar low-resource settings.

### 2.6. Data Management and Analysis

Audio recordings were transcribed verbatim in English by the same researchers who conducted the interviews. Inductive thematic analysis was conducted using MAXQDA (version 2025). Codes were developed iteratively and organized into subthemes and overarching themes. Two researchers independently coded transcripts, using a comprehensive codebook that was developed after a full round of reading the entire manuscript, with discrepancies resolved through consensus (Supplementary File S1). Themes were supported with illustrative quotes to ensure authenticity and depth.

### 2.7. Ethical Considerations

Ethical approval was obtained from the Armauer Hansen Research Institute Institutional Ethics Review Committee with reference number PO-040/25. Written informed consent was obtained from all study participants, who were assured of confidentiality, voluntary participation, and the right to withdraw without repercussions. Data were securely stored and accessible only to the research team. All measures ensured ethical compliance and promoted candid discussion regarding AI-powered ECG interpretation CDSS adoption in routine cardiovascular care.

## 3. Results

### 3.1. Characteristics of Study Participants

A total of 31 healthcare providers working in cardiovascular disease management from 9 referral and specialized hospitals participated in this study, of whom most (28) were males, with an average age of 35.6 years. Regarding the specialties, most (13) were either cardiologists or internists, and around half of them (14) were second-degree holders and served in cardiovascular disease care for an average of 6.4 years (Table 1).

**Figure 1:**
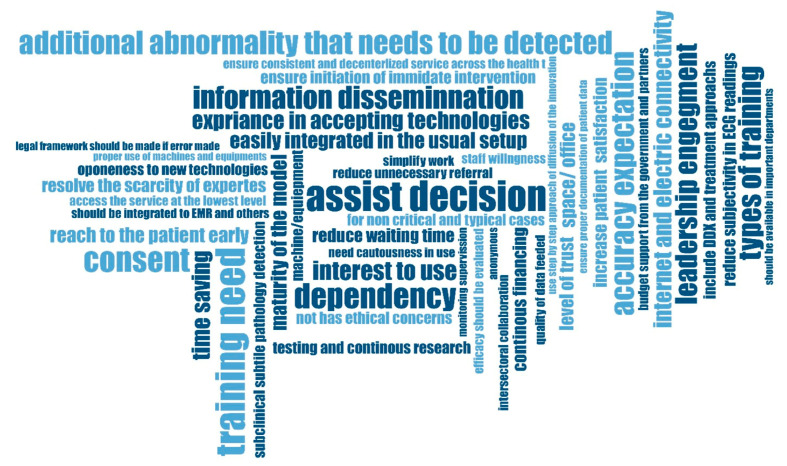
Code cloud structure generated from the data analysis in the study.

Following an exploration of the entire transcription regarding healthcare providers’ perceptions of AI-powered ECG interpretation CDSS, 60 codes were generated and grouped under 10 subthemes, which are further categorized into 6 main themes (Figure 1). The perceived benefits of AI-powered ECG interpretation CDSS, context to develop trust, and integration into the current clinical workflow, ethical concerns, levels of functionality, adoption determinants, and suggested recommendations for better functioning and sustainability were the main themes that emerged from the analysis.

### 3.2. Healthcare Providers’ Perception Regarding the Benefit of AI-Powered ECG Interpretation CDSS

According to our study participants, the key benefits of AI-powered ECG interpretation were highlighted for real-world clinical practice. To ensure coherence and logical elaboration, these perceived benefits were categorized into those related to strengthening the health system and those directly relevant to frontline CVD patient care.

#### 3.2.1. Perceived Benefits of AI-Powered ECG Interpretation CDSS in Strengthening the Health System

Our study participants highlighted the significant role that AI-powered ECG interpretation CDSS can play in strengthening the health system, particularly in resource-limited settings. They emphasized that the tool enables ECG services to be accessed even at the lowest levels of care, simplifying clinical workflows and helping to overcome the persistent scarcity of experts for paper-based ECG interpreters. Furthermore, the technology was perceived as a means to improve the overall efficiency and quality of care by reducing dependence on limited specialists and supporting more equitable access to essential cardiovascular diagnostic services across different levels of the health system.

“*The AI-powered ECG interpretation clinical decision support system may be especially important in non-specialized healthcare facilities. In tertiary hospitals, cardiologists, cardiac nurses, and fellows are available, making the detection of emergency signs more reliable. In contrast, general hospitals may lack these specialists, which can make timely detection of critical signs more challenging. Therefore, implementing an AI-powered ECG system with a low error rate would be particularly valuable in these settings*.”

(A 36-year-old male ICU head nurse)

“*Since the world is moving into the era of AI (in fact, we are now working on X-ray AI), and we don’t have enough cardiologists in remote areas, without needing high-level experts, in a short time we can deliver clinical service*.”

(A 41-year-old, male internist)

Participants also noted that AI-driven ECG interpretation promotes consistent, decentralized CVD care delivery across health tiers while reducing subjectivity in ECG readings. They explained that the technology helps standardize ECG interpretation by applying uniform algorithms, thereby minimizing variations that may arise from differences in clinicians’ experience and expertise.

“*If the AI system is properly developed, it can not only minimize effort but also help unify different expert interpretations. Often, readings vary from one expert to another; some findings might be overlooked or considered unimportant, even though they are significant. So, if we feed it the data properly, AI can summarize the results in a short time and in a simple way. Therefore, I believe it is useful in all these aspects.*”

(A 35-year-old male internist)

Moreover, the tool has the capacity to detect subclinical and subtle cardiac pathologies, save time in clinical decisions, and reduce unnecessary referrals. Collectively, these perceived benefits illustrate how AI-powered ECG interpretation CDSS could save time, improve service quality, and contribute to a more efficient, equitable, and responsive health system.

“*Second, there are small findings humans cannot read and can be identified by the AI powered ECG. It also picks sub clinical pathologies that look normal. There is no question AI powered ECG interpretation is important*.”

(A 40-year-old, male cardiologist)

“*It has also helped reduce unnecessary referrals, ensuring that patients who can be managed at our hospital receive care locally, while those needing advanced management are appropriately referred. As a result, the tool has improved diagnostic accuracy, enhanced patient outcomes, ensured appropriate referrals*.”

(A 27-year-old male general practitioner)

#### 3.2.2. Perceived Benefits of AI-Powered ECG Interpretation CDSS to Patients at Point-of-Care

Healthcare providers emphasized that AI-powered ECG interpretation CDSS holds substantial value for patients at the point-of-care in service delivery. They reported that this technology can enhance the accuracy and completeness of patient data through proper documentation while significantly reducing waiting time. In addition, study participants highlighted that improved documentation and digital storage of ECG results can support better patient follow-up and continuity of care, as records can be easily retrieved and reviewed during subsequent visits.

“*It will store the patients’ data, and it will also compare the previous patient diagnosis with the recent diagnosis, which is very important. Already we have discussed a lot about it*.”

(A 36-year-old male critical care specialist)

Study participants highlighted that AI-supported ECG interpretation allows patients to access diagnostic services earlier, which in turn facilitates the timely initiation of appropriate interventions. By streamlining the diagnostic process and accelerating clinical decision-making, the technology was perceived to greatly improve patients’ overall experience and satisfaction. Collectively, these insights reflect the potential of AI-powered ECG interpretation CDSS to strengthen patient-centered care at the point-of-care.

“*Physicians are often absent in remote areas. In these settings, AI-powered ECG interpretation clinical decision support system could be extremely helpful for early detection and diagnosis of patients’ conditions. For example, in cases of myocardial infarction, timely interventions like PCI or CABG are critical. AI-powered ECG interpretation clinical decision support system could enable rapid identification and referral of patients to facilities where these treatments are available*.”

(A 36-year-old male anesthesiologist)

“*Time is one of the limiting factors in improving patients’ satisfaction. So, the AI-powered ECG interpretation clinical decision support system might save time, and facilitate the probability of getting the appropriate care; it could have a positive impact on patients’ satisfaction*.”

(A 32-year-old male internist)

### 3.3. Contexts to Develop Trust and Integrate into the Current Clinical Workflow

Contexts to develop trust and to integrate the current clinical workflow were another key area/theme that healthcare providers deeply explored. This theme reflects two interconnected areas: the contexts required to build trust in the technology and the contexts needed to ensure its smooth integration into existing clinical workflows. Participants emphasized that both trust-building and workflow alignment are foundational to embedding AI-powered ECG interpretation CDSS sustainably within the healthcare system.

#### 3.3.1. Contexts to Develop Trust in AI-Powered ECG Interpretation CDSS

Healthcare providers highlighted critical contexts that shape their trust in AI-powered ECG interpretation CDSS. Central to this trust is the expectation that the technology must demonstrate high accuracy, supported by reliable and high-quality data inputs. Participants emphasized that confidence in AI-powered ECG interpretation CDSS largely depends on the perceived reliability of the results it generates. They noted that if the system consistently produces accurate interpretations based on quality data, healthcare providers would develop more trust.

“*Accuracy is essential in the health sector; the system must interpret the data provided to it correctly. The acceptable margin of error should be very small, ideally around 0.01, or 99% accuracy. If the accuracy falls below this level, the system can still be used as a supportive tool to guide*.”

(A 41-year-old male ICU head nurse)

Study participants stressed that the clinical efficacy of AI-generated interpretations should be rigorously evaluated before routine use, emphasizing the need for evidence-based validation. They also noted the need for cautious and responsible application of the tool, particularly in settings where misinterpretation could have serious consequences.

“*AI by itself cannot treat a patient. AI helps. Expertise is important. Its efficacy must be evaluated. Sometimes AI can be misleading. Accuracy must be evaluated. Probably, research could be done to check and compare with the expert evaluation of the ECG for accuracy*.”

(A 39-year-old female internist)

“*Follows up. Like other technologies and sciences, there could be errors in AI technologies as well. There might be faults in some areas, so it needs curiosity. I am confident in utilizing AI, but as I told you before, it needs curiosity, deep view, research, and experience*.”

(A 38-year-old male anesthesiologist)

Overall, the level of trust among frontline users is strongly influenced by how well the system performs, the quality of the data it processes, and the assurance that it has been thoroughly assessed for safety and effectiveness.

#### 3.3.2. Contexts to Integrate AI-Powered ECG Interpretation CDSS into the Current Real-World Clinical Workflow

Healthcare providers identified contextual factors that determine how smoothly AI-powered ECG interpretation CDSS can be incorporated into everyday clinical practice. Some emphasized that the system can be easily integrated into the usual clinical setup and with minimal manpower. However, most of the study participants pointed out that for successful integration, the technology should be supported by a mature and reliable AI model and be made available in key service departments where ECG interpretation is essential. Furthermore, seamless integration with existing electronic medical records (EMRs) and digital systems was highlighted as essential for ensuring efficiency and continuity of care.

“*As I mentioned before, the AI must be well-trained while taking into account our culture, norms, patients, and healthcare providers. I have emphasized this earlier as well. We can achieve effective results if it is trained with our specific context*.”

(A 34-year-old internist provider)

“*Due to our country’s current situation, ECG machines are usually available in the emergency department and ICU, but ideally, they should be accessible in all wards. For example, for a diabetic patient admitted to a ward and on insulin, we would be able to detect electrolyte imbalances if the patient is monitored with an ECG. In resource-limited countries, large-scale distribution may not be immediately feasible and would need to be done gradually*.”

(A 37-year-old male emergency care nurse)

“*As I mentioned earlier, if this AI technology is introduced together with the electronic medical recording system, I believe it will be good for its sustainability, and its advantages will also increase. Implementing AI-powered ECG interpretation clinical decision support system without an electronic medical recording system may not be sustainable. So, integrating them together is the better.*”

(A 38-year-old male cardiologist)

Study participants also noted that while AI is useful, it lacks the ability to account for emotional and other intrinsic patient factors and should preferably be applied to non-critical cases during early stages of implementation. Many respondents raised a cautious and phased implementation approach should be followed, suggesting that CDSS for AI-powered ECG interpretation be initially applied in non-critical or supportive roles; then, its reliability and contextual adaptability will be further evaluated.

“*Even though AI learns, we do not believe it can replace humans. Our brain makes decisions after taking different factors into account. The first aspect is the emotional aspect, which is lacking in many AIs. Emotional intelligence is currently being incorporated, but most AIs still lack it, they only have artificial intelligence. Another aspect is that the social, economic, and spiritual conditions of patients must also be involved in decision-making. Moreover, when you interpret results, you don’t do so just by looking at papers; you also observe the patient*.”

(A 34-year-old male internist)

### 3.4. Ethical Issues and Level of Functionality Regarding AI-Powered ECG Interpretation CDSS

Under this theme our study participants described about ethical acceptability and their expectations regarding the level of functionality of the AI model and related challenges.

#### 3.4.1. Ethical Issues Concerning the Use of AI-Powered ECG Interpretation CDSS

While most healthcare providers who participated in this study believed that the technology does not inherently pose major ethical concerns, they agreed that establishing proper safeguards is vital for ethical and trustworthy implementation. Some healthcare providers raised important ethical considerations that must be addressed for the responsible use of AI-powered ECG interpretation CDSS. They emphasized that maintaining the anonymity and confidentiality of patient data entered into the model is essential to safeguard privacy.

“*ECG result must be confidential just like any other patient information. Blood type, blood pressure, heart beat, name, etc. patient information must stay confidential. Result generated by AI powered ECG is no different from lab result or any patient information. It will be part of the whole patient care. When caring for patient we keep their information confidential whether we use hard copy or electronic health record system. The same goes for AI-powered ECG interpretation clinical decision support system*.”

(A 46-year-old male cardiologist)

Some participants also noted that informed verbal consent should be obtained before using AI-assisted diagnostic tools, as the normal clinical procedure. Additionally, the need for a clear legal framework was highlighted, particularly to define accountability in cases where AI-generated interpretations may lead to errors. Participants stressed that well-defined regulatory and ethical guidelines are necessary to clarify roles and responsibilities among healthcare providers, health institutions, and technology developers to ensure patient safety, protect healthcare providers, and support the responsible integration of AI technologies into routine clinical practice.

“*Since this is new technology, obtaining consent is necessary because patient data is involved. We need to ask for patient consent explicitly by saying, “We will be feeding your data into the machine. What do you think?*”

(A 48-year-old male cardiologist)

“*There might be some ethical concerns related to medical ethics, such as who will be accountable if a medical error occurs because of an intervention made based on the device’s interpretation. There might be complex issues, but errors can also be made by humans. I’m not against the AI-powered device, but to avoid such issues, some legal framework should be developed before implementation*.”

(A 34-year-old male general practitioner)

#### 3.4.2. Expected Level of Functionality of AI-Powered ECG Interpretation CDSS

Healthcare providers expressed clear expectations regarding the appropriate level of functionality for AI-powered ECG interpretation CDSS. They emphasized that the technology should serve primarily as an assistive/supportive tool, rather than replace the clinician in decision-making.

“*Ultimately, the use of this tool should never override the physician’s clinical judgment. For instance, there may be situations where my assessment of a patient differs from the tool’s suggestion, as medicine is a complex field that relies heavily on a physician’s intuition and judgment. I would not want to lose that intuition or depend entirely on the tool; rather, I see it as a supportive aid that should not compromise my decision-making*.”

(A 35-year-old male internist)

“*The final decision will always be made by the healthcare provider; the AI tool is intended only as a supportive aid. Patients do not come to the hospital solely for an ECG, they often have other health issues as well. Therefore, I do not believe that the AI system will replace the role of healthcare providers; rather, it will serve to assist*.”

(A 32-year-old female ICU nurse)

Study participants noted concerns that relying on AI as a full decision-maker could inadvertently create dependency, potentially diminishing clinicians’ ongoing professional development and reducing their day-to-day preparedness for clinical practice. Instead, they stressed that AI should complement human expertise by enhancing accuracy and efficiency while preserving the clinician’s central role in interpreting findings and making final decisions. This balanced expectation reflects the desire to harness the benefits of AI without compromising the growth and autonomy of healthcare providers.

“*When we see the drawback, it decreases our critical thinking. Because it is the survival of the fitness. Our brain will adapt simple things and it will avoid hardships. The AI powered ECG interpret things simply, but in order to interpret by your own, you might need to read days, weeks, and months. So progressively the brain prefers the simpler one and it will adapt it though out time. So, it decreases the judgmental thinking of physicians and they become dependent on AI powered ECG, and it will also affect their autonomy*.”

(A 34-year-old male internist)

### 3.5. Adoption Determinants of AI-Powered ECG Interpretation CDSS

Healthcare providers identified multiple *adoption determinants of AI-powered ECG* interpretation CDSS that influence the effective and sustainable adoption of the technology in their respective health facilities. Their perspectives revealed three interconnected areas shaping adoption: material resource–related determinants, human resource–related determinants, and governance and administrative challenges. These subthemes reflect how the availability of appropriate equipment and infrastructure, the readiness and capacity of healthcare workers, and the presence of supportive policies and institutional frameworks all play crucial roles in determining the feasibility of AI-powered ECG interpretation CDSS implementation.

#### 3.5.1. Material Resource-Related Determinants

Healthcare providers highlighted material resource–related determinants that significantly influence the feasibility of adopting AI-powered ECG interpretation CDSS. They emphasized that reliable internet and electricity connectivity are foundational requirements, as interruptions in either can limit system functionality and compromise timely patient care.

“*Since the system requires internet connectivity, a reliable and high-speed connection is essential. Additionally, a stable electricity supply must be ensured, as interruptions could affect the function of the technology*.”

(A 36-year-old male general practitioner)

The availability of appropriate machines and equipment, such as computers, was also noted as essential to the operation of the AI tool. In addition, participants pointed to the need for adequate physical space or office arrangements to properly house the devices and facilitate workflow.

“*Resource shortages exist. For example, uploading ECGs requires USBs, internet cables, computers, and printers, as well as paper. Previously, when paper was unavailable, results were photographed. The AI-powered ECG interpretation clinical decision support system generates results as XML/PDFs, which helps*.”

(A 35-year-old male ICU nurse)

“*As you can see, our current ICU is quite narrow. Although it contains ECG and ultrasound machines, the space is limited, and we are planning to improve it. A new ICU facility is currently under development. When the AI-powered ECG interpretation clinical decision support system becomes available in our hospital, it will likely require a separate and spacious room*.”

(A 38-year-old male anesthesiologist)

#### 3.5.2. Human Resource-Related Determinants

Healthcare providers identified human resources–related factors that shape the successful adoption of an AI-powered ECG interpretation CDSS. Central to these determinants is the willingness of staff to engage with the technology, as openness to innovation strongly influences uptake. Participants also noted that prior experience with digital tools and technology acceptance plays a major role.

“*When new things become applied, the implementation might not be as easy as expected. Thus, the role and the acceptance of the technology by the physician have to be considered, and administrators has to consider the common understanding of physicians during the decision-making process to apply new technologies*.”

(A 32-year-old male internist)

In addition, study participants emphasized the need for targeted training for healthcare providers who had frequently used the tool to ensure competent operation, highlighting both the significance of structured capacity building, which mainly used skill-improvement training formats such as simulation and onsite practical demonstration.

“*First, training is essential. From setting up the machine to reading the results. Is the result the right patients? How is patient data entered? How is it read? What must be done when the reading does not correlate with patient symptoms? So, health workers who will be involved such nurses, physicians must be trained*.”

(A 42-year-old male cardiologist)

“*Face to face or practical training is better. Using actual patient and ECG. Comparing what physician reads with AI generated interpretation. So practical training is better*.”

(A 40-year-old male cardiologist)

#### 3.5.3. Governance and Administration-Related Challenges

Healthcare providers pointed out governance and administrative challenges that could influence the successful implementation of AI-powered ECG interpretation CDSS in their respective facilities. They highlighted the critical role of strong leadership engagement for technology acceptance.

“*Attention and support from hospital leadership are crucial during implementation to ensure the technology effectively improves service delivery. It is important to approach new technologies with an open mind.*”

(A 36-year-old female ICU nurse)

Study participants also emphasized the need for sustainable budgeting and financing, as inconsistent or inadequate resource allocation could hinder system maintenance, upgrades, and long-term functionality.

“*Challenges may arise due to budget constraints; as new technologies usually require significant investment. Leadership acceptance depends largely on budget considerations*.”

(A 38-year-old male internist)

### 3.6. Recommendation for Better Functioning and Sustainability of AI-Powered ECG Interpretation CDSS

Healthcare providers suggested a wide range of recommendations aimed at ensuring the effective functioning and long-term sustainability of AI-powered ECG interpretation CDSS. They emphasized the importance of proper use and maintenance of digital ECG machines and equipment and the need for strong budgetary support from the government and partners as part of intersectoral collaboration.

“*Since it is a new technology, it can be very expensive, so related to the budget, it may be difficult to provide these machines; therefore, aids may be needed. Most hospitals need to have this machine and related accessory equipment like computer, internet for the machine. Mostly, the sustainability issues for new technologies like this are the dysfunctionality of the equipment. For this issue, trained biomedical professionals in the institutions are needed to maintain*.”

(A 29-year-old male general practitioner)

Study participants highlighted their perceived benefits of openness among health workers and institutions in embracing new digital health technologies, along with adopting a step-by-step approach to the diffusion of innovation and learning from past experiences to support smooth integration.

“*We need to remain open to new technologies, especially in the health sector, as embracing innovation is essential for improving the healthcare system*.”

(A 34-year-old male ICU nurse)

“*A phased approach is necessary. When new technology comes, if we take a head-on approach, it may fail. Step by step, starting with facilities ready for change, would make it effective*.”

(A 46-year-old male cardiologist)

Clear and continuous information dissemination, ongoing testing and research, and regular monitoring and supervision to guide improvements were also identified as key strategies to strengthen system performance.

“*It should be tested. Research is needed. Its practicality must be thoroughly evaluated. There should be a project on Quality improvement. Hospital administration must understand what is required and supervise the process*.”

(A 46-year-old male cardiologist)

In addition, healthcare providers underscored the need to expand the AI model’s training to include more cardiac abnormalities, differential diagnoses, and basic management approaches to enhance clinical usefulness. Collectively, these recommendations reflect a comprehensive vision for optimizing and sustaining the role of AI-powered ECG interpretation clinical decision support in the health system.

“*In addition to standard measurements like heart rhythm, bundle branch blocks, and segment changes, AI should evaluate P-wave morphology, PR intervals, T-wave changes, QT intervals, and other parameters essential for ICU and emergency management. Analysis of electrolyte imbalances, which can be identified by ECG, should be included. This is what I expect from the device*.”

(A 36-year-old male internist)

“*Since AI is involved, I hope that in the future it will also be able to suggest management options. At the moment, the system is mainly used for interpretation and does not provide possible treatment recommendations. When we refer to interpretation, it essentially means that the system makes a diagnosis. It would be beneficial if, over time, the technology could evolve to include treatment suggestions following interpretation*.”

(A 34-year-old male ICU nurse)

## 4. Discussion

This study explored healthcare providers’ perceptions and multi-level determinants influencing the adoption of AI-powered ECG interpretation CDSS at the point-of-care in Ethiopia. Overall, study participants expressed strong optimism regarding the technology’s potential to strengthen cardiovascular care delivery, particularly in resource-limited and non-specialized healthcare settings. These findings align with growing evidence that AI-enabled diagnostics can mitigate workforce shortages and improve clinical decision-making in low- and middle-income countries [6,15]. Grounded in the diffusion of innovation framework, this optimism reflects the perceived relative advantage and comparability of the technology, underscoring its potential for structured scale-up.

Healthcare providers perceived AI-powered ECG interpretation CDSS as a critical tool for strengthening health system efficiency. They emphasized its role in decentralizing ECG interpretation skills, reducing subjectivity, detecting subtle abnormalities, and minimizing unnecessary referrals. Such system-level benefits are consistent with prior studies demonstrating AI’s role in enhancing diagnostic equity and continuity of care [16,17]. Within the Health Technology Acceptance Model (TAM), these perceptions reflect perceived usefulness, which strongly influences adoption intentions. In Ethiopia’s context of limited specialized healthcare providers and infrastructure, these findings highlight the importance of positioning AI-powered ECH interpretation CDSS as a system-strengthening intervention.

From a patient-centered perspective, an AI-powered ECG interpretation CDSS was seen to improve access, reduce waiting times, and support earlier diagnosis and referral, especially in remote areas. These benefits are highly relevant in time-sensitive cardiac conditions where delays worsen outcomes [18]. Interpreted through access-to-care frameworks, these perceptions highlight improvements in availability, accessibility, and timeliness [19]. They also align with the diffusion of innovation concept of observability, as patients directly experience the benefits of earlier diagnosis and referral. This complements existing literature on AI-powered ECG interpretation CDSS [20,21] and suggests that in Ethiopia, where cardiovascular care services are concentrated in urban hospitals, AI-powered ECG interpretation CDSS can alleviate patient concerns and enhance satisfaction.

Trust emerged as a foundational requirement for adoption, with healthcare providers emphasizing diagnostic accuracy, data quality, and validation against expert interpretation. Concerns about misleading outputs and inappropriate reliance reflect a cautious stance. The findings align with global literature on trust in AI-powered ECG interpretation CDSS [22,23]. With TAM, trust relates to perceived ease of use and perceived risk, both of which shape acceptance. In Ethiopia, structural challenges such as limited digitization and fragmented health data records further condition trust, highlighting the need for transparent performance evaluation and evidence-based deployment.

Integration into clinical workflows was viewed as feasible but dependent on contextual adaptation, EMR interoperability, and phased implementation. Healthcare providers stressed that AI-powered ECG interpretation CDSS should complement, not replace, clinical judgment, particularly in emergency and ICU settings. This balanced view was also reflected by previously published evidence emphasizing professional autonomy [24,25]. From a diffusion of innovation perspective, this reflects trialability, where phased implementation allows providers to test AI in practice while maintaining clinical oversight. Regulatory clarity on liability and accountability remains essential in Ethiopia’s evolving governance landscape.

Adoption determinants extended beyond technology to infrastructure readiness, workforce capacity, leadership engagement, and governance frameworks. Reliable electricity, internet access, training, budgeting, and accountability were seen as prerequisites for sustainability. Similar findings have been reported in other digital health adoption studies [26,27]. Conceptually, these determinants reflect the broader innovation system, were organizational readiness and leadership support influence adoption trajectories. Coordinated national frameworks and context-specific implementation strategies—including phased rollout, pilot testing, and continuous capacity building—are critical for sustainable adoption.

### Limitations

This study has limitations that should be considered when interpreting the findings. The sample size of 31 study participants was determined by information saturation, consistent with qualitative research standards, though it may limit the breadth of perspectives. A gender imbalance, with most participants being male, could have influenced the range of views represented. Social desirability bias cannot be excluded, as growing institutional interest in digital health and AI may have shaped favorable responses. Patient perspectives were not included, which are important for a more comprehensive understanding of adoption in real-world settings.

## 5. Conclusions

Healthcare providers in Ethiopia perceive AI-powered ECG interpretation CDSS as a highly promising tool for improving cardiovascular care delivery, efficiency, and equity across health system levels. Its potential to support timely diagnosis, reduce workload, and enhance patient-centered care is widely recognized, particularly in non-specialized and resource-constrained settings. High model accuracy, contextualized training, workflow integration, ethical safeguards, and strong governance support are critically needed for sustainable adoption. A phased, evidence-based implementation that positions AI as a decision-support tool rather than a replacement for clinical judgment will be essential to maximize benefits while preserving healthcare providers’ professional autonomy and patient safety.

## Figures and Tables

**Table 1:** Characteristics of study participants included in the study, 2025.

Variable	Category	Value
Age in years	Average	35.6
Sex	Male	28
	Female	3
Specialty	Cardiac nurses	10
	Anesthesiologists	2
	General practitioners	6
	Cardiologists/internists	13
Educational level	First degree	9
	Second degree	14
	Third degree and above	8
Experience in CVD care (years)	Average	6.4

## Data Availability

All relevant data are within the manuscript and its Supplementary Materials File.

## Supplementary Materials

The following supporting information can be downloaded at: https://docs.google.com/document/d/19eGAjnVNKopxj-FOfD4ICRCvdN_asdxu/edit?usp=drive_link&ouid=107046706163683278123&rtpof=true&sd=true (accessed on 25 November 2025) Supplementary File S1: Codebook developed for the study.

## Abbreviations

The following abbreviations are used in this manuscript:

AI	Artificial intelligence
AHRI	Armaur Hansson Research Institute
CDSS	Clinical decision support system
CVD	Cardiovascular disease
ECG	Electrocardiography
IDI	In-depth interview
LMICs	Lower and middle-income countries
NCD	Noncommunicable disease
WHO	World Health Organization
